# Structure-Informed Design of an Ultra Bright RNA-activated Fluorophore

**DOI:** 10.21203/rs.3.rs-4750449/v1

**Published:** 2024-08-05

**Authors:** John S. Schneekloth, Mo Yang, Peri Prestwood, Luiz Passalacqua, Sumirtha Balaratnam, Christopher Fullenkamp, Winston Arney, Kevin M. Weeks, Adrian Ferre-D’Amare

**Affiliations:** NCI; National Cancer Institute; National Cancer Institute; National Heart, Lung, and Blood Institute; National Cancer Institute; National Cancer Institute; University of North Carolina, Chapel Hill; University of North Carolina at Chapel Hill; National Heart Lung and Blood Institute

## Abstract

Fluorogenic RNAs such as the Mango aptamers are uniquely powerful tools for imaging RNA. A central challenge has been to develop brighter, more specific, and higher affinity aptamer-ligand systems for cellular imaging. Here, we report an ultra-bright fluorophore for the Mango II system discovered using a structure-informed, fragment-based small molecule microarray approach. The new dye, Structure informed, Array-enabled LigAnD 1 (SALAD1) exhibits 3.5-fold brighter fluorescence than TO1-Biotin and subnanomolar aptamer affinity. Improved performance comes solely from alteration of dye-RNA interactions, without alteration of the chromophore itself. Multiple high-resolution structures reveal a unique and specific binding mode for the new dye resulting from improved pocket occupancy, a more defined binding pose, and a novel bonding interaction with potassium. The dye notably improves in-cell confocal RNA imaging. This work provides both introduces a new RNA-activated fluorophore and also a powerful demonstration of how to leverage fragment-based ligand discovery against RNA targets.

## INTRODUCTION

The development of RNA aptamers that fluoresce when bound to small molecule dyes has shown great potential in the field of RNA imaging.^1, 2^ Binding between dyes and RNA aptamers significantly enhances fluorescence yielding excellent signal to noise ratios. Many different fluorogenic RNA aptamers have now been developed, including the malachite green aptamer, Spinach, Broccoli, Mango, Corn, and Pepper, thioflavin T-based systems,^3^ PNA-based probes,^4^ and related variants.^5, 6, 7^ More recently, fluorogenic DNA aptamers have been reported.^8^ In general, these aptamers bind to a variety of small molecule dyes, some inspired by the green fluorescent protein chromophore, to induce greatly enhanced fluorescence of the dye.^9^ Aptamers have been developed that fluoresce across a wide range of emission wavelengths ranging from 500 nm to 660 nm.^6^ Fluorogenic aptamers have been used in diverse cellular imaging studies, including multi-color RNA imaging and single-molecule spectroscopy.^10, 11, 12, 13, 14^ In addition to their use as imaging agents, fluorogenic aptamers have been leveraged to create sensors.^15, 16^ The fact that these aptamers are genetically encodable, coupled with flexibility in choicd of excitation-emission wavelengths makes them powerful tools for diverse applications.

Among the above-mentioned RNA aptamers, Mango is a well characterized system that binds tightly to thiazole orange (TO) and related dyes.^17^ To date, four generations of Mango aptamers have been developed, all exhibiting low nanomolar affinities to TO derivatives including TO1-Biotin and TO3-biotin.^18^ In-depth structural studies including X-ray crystallography have revealed that the aptamers fold into a complex G-quadruplex-containing structure and accomplish turn-on fluorescence by constraining the TO fluorophore into a planar conformation.^17, 19, 20, 21^ The Mango II aptamer contains a well-defined but plastic pocket capable of binding TO derivatives in multiple orientations. To date, most work on the Mango system has focused on evolution of improved aptamers,^22, 23^ development of fluorogenic transcripts for live cell or single molecule imaging,^24^ or rational modification of the TO fluorophore to alter excitation/emission profiles.^25^ The discovery of Mango aptamers represents a powerful advance in the development of RNA imaging tools.

The existence of high-quality structural information coupled with uniquely valuable imaging applications makes the Mango aptamers an attractive system for structure-informed ligand design against RNA. Recognition of RNA as an important target for small molecules is increasing.^26, 27, 28, 29^ However, strategies to develop potent, selective small molecule ligands for RNA still lag far behind protein targeting strategies. One approach that has gained some attention is fragment-based design. Fragment-based drug design enables the rapid development of tight and specific small molecule binders for a target from weak but highly specific low molecular weight ligands.^30, 31^ In recent years, fragment-based technologies have been broadly applied to RNA,^32^ including nuclear magnetic resonance (NMR),^33, 34^ equilibrium dialysis,^35^ selective 2’-hydroxyl acylation analyzed by primer extension and mutational profiling (SHAPE-MaP),^36^ surface plasmon resonance (SPR),^37^ mass spectrometry (MS),^38^ and DNA encoded library (DEL)-based screening.^39^ Upon performing a ligandability analysis of the Mango II TO-binding pocket, we reasoned that a fragment-based screening approach could be an appropriate pathway to develop new fluorescent RNA ligands with improved properties.

Here, we report the structure-informed discovery of a new generation of TO-derived fluorophores for the Mango II RNA aptamer using a fragment-based microarray screening strategy. Biophysical analysis of dye- and fragment-RNA interactions revealed non-competitive binding between one identified fragment and TO. By linking the fragment and TO, multiple new dyes were created, with sub-nanomolar binding affinities. Critically, one compound (SALAD1) exhibited 3.5-fold improved brightness relative to TO and TO1-Biotin, both of which are typically used in cellular imaging with Mango aptamers. Structure visualization with four different dye-aptamer complexes revealed a unique binding mode distinct from TO1-Biotin. Photophysical analysis and structure-activity relationship studies revealed a critical combination of functional groups necessary for the observed fluorescence enhancement. Finally, the improved fluorescence yields notable advantages in high-signal confocal cellular imaging. Taken together, this structure-informed approach reveals how fragment-based ligand design, targeted against an RNA aptamer, can lead to notable enhancement of fluorescence and in-cell imaging by constraining the fluorophore in a unique binding pose.

## RESULTS AND DISCUSSION

### High-throughput screening for Mango II RNA-binding fragments

**Figure 1:**
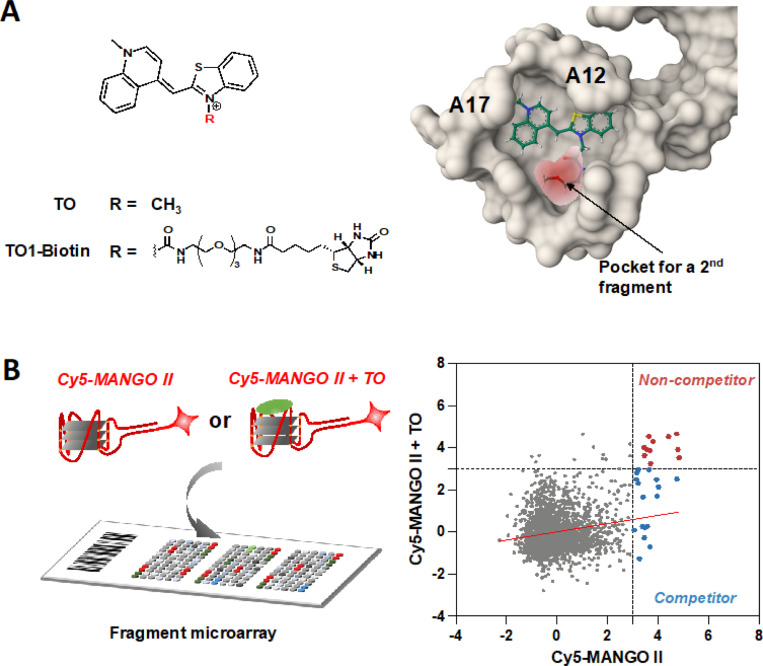
Structure and fragment-binding to the Mango aptamer. (A) Pocket analysis of Mango II RNA aptamer modeled in presence of TO. (B) Fragment microarray-based screening using Cy5-labeled Mango II RNA (250 nM) with/without competing TO (2.5 μM). Replicate screenings were performed for each sample. Z-score comparison of each fragment as a function of incubation conditions (Mango II vs. Mango II + TO). Fragments bind to the Mango II aptamer in both competitive and non-competitive modes.

To develop a novel TO-derived fluorophore, we first analyzed the binding pockets for ligands within the Mango aptamers. The binding modes of TO1-based ligands in all four Mango aptamers were compared qualitatively and using ICM MolSoft Pocketfinder software (Figure S1). Analysis of the co-crystal structure of TO1-Mango II complex (PDB: 6C63) revealed that the ligand binding pocket is hydrophobic and within acceptable volume for recognizing small molecule ligands. When bound to the aptamer, TO1-Biotin stacks on the guanine tetrads and interacts with A12 and A17, while the biotin sidechain was solvent exposed and not resolved in the crystal structure. By docking a structure of TO (which lacks the biotin-bearing sidechain) to the Mango II aptamer, we found that an empty space exists within the pocket. We reasoned that this space could accommodate a separate fragment, potentially linked through the methyl group on TO, that could be leveraged to improve binding (Fig. 1A and S2).

To efficiently identify a TO co-binder, we developed fragment microarrays that could be used to screen tagged Mango aptamers in the presence or absence of the TO ligand. Briefly, a total of 2,214 fragments were curated and purchased from Enamine, all of which contained amine and alcohol groups compatible with array manufacture. Fragments were printed onto isocyanate-modified glass slides based on previously reported methods for small molecule microarray (SMM) fabrication.^40^ In parallel, a screening construct consisting of the Mango II RNA tagged with a poly-A tail was annealed with a Cy5-poly(dT) oligonucleotide. This construct was dissolved in a folding buffer containing140 mM KCl, annealed, and analyzed by circular dichroism (CD) to confirm proper aptamer folding. The screening construct exhibited a maximum at 263 nm and a minimum at 240 nm, consistent with a folded, parallel G4 structure. These features were not observed in LiCl buffer (**Figure S3**). Once proper folding was confirmed, arrays were incubated with the screening construct, and the fluorescence intensity for each spot was quantified (using Z-scores, across two replicates). In parallel, a second screen was performed in the presence of a saturating concentration (10 equivalents) of TO. Pearson correlation coefficient (r) values for Mango II and Mango II + TO assays were 0.81 and 0.84, respectively, confirming a reproducible screen (**Figure S4A**). In contrast, comparing the two different screening results yielded a Pearson correlation of 0.04, indicating that distinct sets of compounds scored as hits in the presence versus absence of TO (Fig. 1B). A total of 30 fragments (**F1-F30**) were identified as hits for Mango II RNA (**Figure S4B**). Binding of 11 fragments was non-competitive with TO (**F1-F11, Figure S5**), while the remaining 19 were identified as competitive (**F12-F30, Figure S6**). We hypothesized that some of the non-competitive hits may bind to the available pocket in the Mango aptamer (Fig. 1A).

### Characterization of Fragment Binding to Mango II

**Figure 2:**
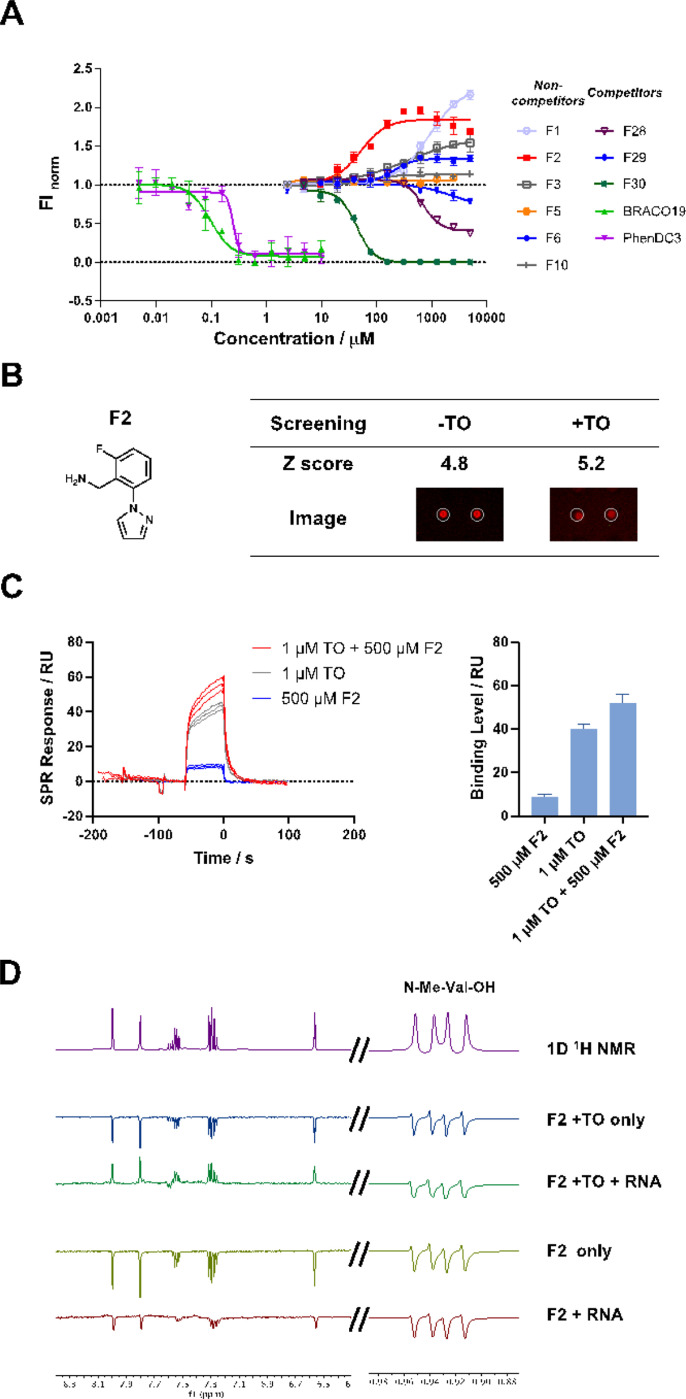
Identification of fragments that co-bind RNA with TO. (A) Fluorescence intensity assay using representative non-competitive fragments (F1-F3, F5, F6, and F10) and competitive fragments (F28-F30) discovered by microarray screening. BRACO19 and PhenDC3, as classical G4-binders, were used as controls in the displacement study. (B) Chemical structure of F2 and SMM screening results (Z-scores and spot images). (C) SPR binding assay for injecting TO, F2, and TO + F2 solutions. Three replicate sensorgrams are shown for each condition. (D) WaterLOGSY NMR assays demonstrating F2 binding with Mango II RNA in presence or absence of TO.

For the 11 fragments showed non-competitive binding behavior with TO by SMM, we explored whether any fragments impacted ligand fluorescence. We performed a fluorescence intensity assay by titrating fragments into a solution containing the TO-Mango II complex. Titrations contained a TO concentration of 500 nM so that binding to the Mango II pocket (K_D_ = 100 nM) was fully saturated. Fluorescence was measured as a function of fragment concentration. Six non-competitive fragments **(F1, F2, F3, F5, F6, and F10)** significantly enhanced the TO fluorescence (ranging from 5–116%), while the other five showed weak or no effects (Fig. 2A). Several fragments (**F1-F3**) capable of enhancing fluorescence contained similar structural chemotype (**Figure S7**). In addition, competitive binders, including three representative fragments (F28, 29 and 30) and two G4 stacking ligands (BRACO19 and PhenDC3), were also tested and indeed showed competition in fluorescence assay, emphasizing our ability to identify co-binding fragments. Among all the fragments, **F2** showed the most promising fluorescence enhancement behavior with an EC_50_ value of 52 ± 19 μM and 95% improvement in fluorescence intensity (Fig. 2A and S7), along with high Z scores (Fig. 2B) and was selected for further study. **F2** itself is not fluorescent, emphasizing that the fragment enhanced the fluorescence of TO itself (**Figure S8**). Together, these observations both validate the ability of SMMs to identify noncompetitive ligands, and also remarkably revealed that noncompetitive and non-covalently binding ligands can enhance the fluorescence of TO.

### Characterization of F2 as a co-binder with TO to Mango II RNA

Binding of **F2** to the TO-Mango II complex was characterized by multiple approaches. Surface plasmon resonance (SPR) was performed using polyA-containing Mango II RNA annealed a with biotinylated poly(dT) oligo, immobilized on a streptavidin surface. Injection of **F2** (500 μM) or TO (1 μM) yielded binding signals of 10 ± 1 and 41 ± 2 response units (RU), respectively. Injecting both TO and **F2** resulted in an observed binding level of 52 ± 4 RU, roughly equivalent to the sum of the response observed for individual components (Fig. 2C), and thus confirming co-binding. Fitting titrations of **F2** showed a K_D_ of 700 ± 260 μM. In a parallel experiment, samples were pre-equilibrated with saturating (500 nM) TO and then titrated with **F2**. In the presence of TO, the dissociation constant remained similar (K_D_= 450 ± 120 μM), indicating that TO binding to the RNA does not significantly impact the KD of **F2**. (**Figure S9**). In parallel, fluorescence titrations were used to confirm the observation that **F2** binding does not influence the K_D_ of TO for the aptamer. (**Figure S10**).

Binding of **F2** was also evaluated using water ligand observed gradient spectroscopy (waterLOGSY) NMR. Here, positive phasing of the ligand peaks identifies a binding interaction (Fig. 2D). Under these conditions, **F2** only bound to Mango II RNA in the presence of TO. In contrast, omitting other components (TO or RNA) led to the negative-phasing NMR spectrum, indicating no interaction. Together, these results confirm that **F2** occupies an RNA binding site distinct from TO.

### Linking TO and F2 yields a dye with enhanced fluorescence

**Figure 3:**
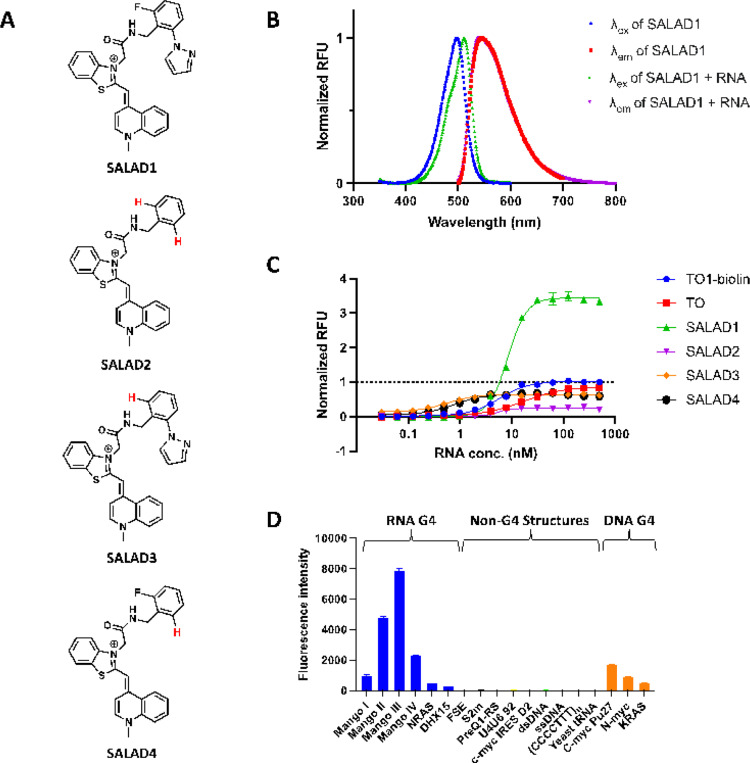
Linked fluorescent probes and their photophysical properties. (A) Structures of designed fluorescent probe and analogues. (B) Excitation (λex) and emission (λem) curves of SALAD1 with and without Mango II. (C) Fluorescence intensity assay comparing thiazole orange (TO), TO1-Biotin, and designed analogues. Data are normalized to TO1-Biotin. (D) Selectivity profile comparing fluorescence of SALAD1 (40 nM) in the presence of representative nucleic acid structures.

Encouraged by the observation that non-covalently bound **F2** enhanced the fluorescence of TO, we designed and synthesized new fluorescent probes by linking TO with **F2** and related compounds. We developed a four-step route to synthesize a new dye, consisting of **F2** linked to TO through an amide linker adjacent to the benzothiazole ring of TO (Fig. 3A, **Supplementary Methods**). The new ligand, Structure-informed, Array-enabled LigAnD, was named SALAD1. In addition, we synthesized three additional analogs lacking functional groups on the fragment benzyl ring—one lacking the fluorine (SALAD3), one lacking the pyrazole ring (SALAD4), and another lacking both groups (SALAD2) (Fig. 3A, **Supplementary Methods**).

Relative to TO (λ_ex_ = 510 nm, λ_em_ = 533 nm) and TO1-Biotin (λ_ex_ = 510 nm, λ_em_ = 535 nm), SALAD1 has a similar but slightly red-shifted excitation and emission profile (λ_ex_ = 511 nm, λ_em_ = 540 nm) (Table 1, Fig. 3B). SALAD1 also displayed a slightly larger Stokes shift of 29 nm relative to TO (22 nm) and TO1-Biotin (25 nm). Similar excitation and emission profiles were observed for the other analogs (**Figure S11**). Relative fluorescence intensities of the dyes were compared through a fluorescence intensity assay (Fig. 3C). The SALAD1 compound displayed greater than 3.5-fold brighter fluorescence than TO and TO1-Biotin at high RNA concentrations. Our other analogs showed lower fluorescence intensities than did SALAD1 and TO, emphasizing that all molecular features of **F2** are necessary to enhance fluorescence.

Further photophysical characterization revealed that SALAD1 displays properties similar to existing TO-based dyes (Table 1). When bound to Mango II, SALAD1 shows a 514-fold fluorescence enhancement, compared to 643- and 647-fold turn-on for TO and TO1-Biotin, respectively. The extinction coefficient of SALAD1 (45,422 M^− 1^cm^− 1^) is also comparable to the extinction coefficients for TO (53,784 M^− 1^cm^− 1^) and TO1-Biotin (77,500 M^− 1^cm^− 1^). SALAD3 is the only other analog that displayed similar properties, with a turn-on of 711-fold and an extinction coefficient of 30,235 M^− 1^cm^− 1^. SALAD2 and SALAD4 showed significantly weaker fluorescence enhancement values and lower extinction coefficients indicating that the pyrazole ring plays a critical role for these properties.

Apparent K_D_ values were determined for each compound using dose-dependent fluorescence intensity assays (Fig. 3C, S12, and Table 1). SALAD1 (K_D_^app^ = 0.69 ± 0.1 nM) binds 7.5-fold more tightly to Mango II compared to TO (K_D_^app^ = 5.9 ± 1.4 nM), demonstrating that the new dye had a significantly improved binding affinity to the aptamer. The observed binding affinity is comparable to TO1-Biotin (K_D_^app^ = 0.85 ± 0.2 nM), despite the difference in fluorescence intensities. Intriguingly, SALAD2 (K_D_^app^ = 0.27 ± 0.03 nM), SALAD3 (K_D_^app^ = 0.29 ± 0.02 nM), and SALAD4 (K_D_^app^ = 0.21 ± 0.03 nM) all displayed tighter binding affinities to Mango II in fluorescence intensity assays, indicating that binding affinity and fluorescence intensity are not directly related for this system.

TO is known to bind nonspecifically to nucleic acid structures, limiting its utility in targeted imaging applications.^41^ We assessed the selectivity of SALAD1 by measuring fluorescence when incubated with representative RNA and DNA structures, including several G-quadruplexes (**Table S1**). SALAD1 showed changes in fluorescence intensity in the presence of all four generations of Mango and fluoresced brightest when bound to Mangos II and III (Fig. 3D). In contrast, weaker or no binding was observed to other G4 and with non-G4 nucleic acid structures, indicating selective interactions at relevant concentrations.

### X-ray crystal structures reveal unique binding mode of new dyes

**Figure 4:**
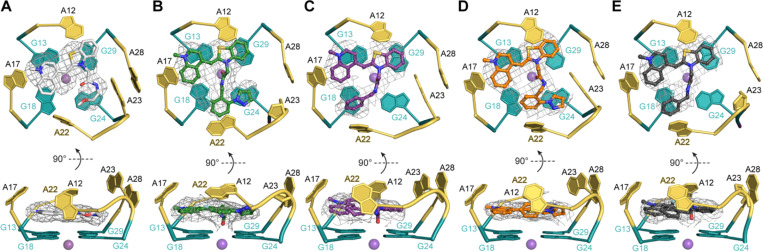
Top and side views of X-ray crystal structures of the Mango II aptamer binding site complexed with (A) TO1-Biotin (PDB: 6C63), (B) SALAD1, (C) SALAD2, (D) SALAD3, and (E) SALAD4. A22 is marked in yellow; purple spheres represent K^+^. Gray meshes depicts |*Fo*| − |*Fc*| electron density map before building the fluorophores, contoured at 1.0 σ.

We determined co-crystal structures of our new fluorophores with the Mango II RNA at 2.85–3.0 Å resolution (**Table S2, Supplementary Methods**). All of the new fluorophores bind the aptamer RNA with a 1:1 stoichiometry, and in the same binding pocket as occupied by TO1-Biotin (**Figure S13**).^19^ Two of the new ligands (SALAD1 and SALAD3) fill the binding pocket to a larger extent than TO1-Biotin (Fig. 4 and S14). The buried solvent-accessible area for SALAD1 and SALAD3 are 590.4 Å^2^ and 594.0 Å^2^, respectively, whereas Mango II buried 529.1 ± 5.1 Å^2^ (average of two well-resolved crystallographically-independent complexes in the structure ± s.d.) In contrast, SALAD2 and SALAD4 have a less extensive RNA interface than TO1-Biotin (burying 514.6 Å^2^ and 514.8 Å^2^, respectively). Regardless of the degree of occupancy of the binding site, the fluorophores exhibit multiple binding poses in all of our co-crystal structures. Thus, the new ligands do not completely resolve the binding-site promiscuity originally noted for the Mango II-TO1-Biotin complex (PDB:6C63).^19^

When TO1-Biotin binds to Mango II, it creates an unoccupied a cavity adjacent to RNA residue A22. This purine nucleotide adopts a similar conformation in the TO1-Biotin complex and in complexes with the new fluorophores, except in the SALAD1 complex. In the SALAD1-containing structure, A22 adopts the *syn-* glycosidic bond conformation (rather than the *anti-* conformation present in all other Mango II complex structures), and the purine base is displaced to the top of the fluorophore (Fig. 4B and S15). Binding of SALAD1 thus results in a substantial rearrangement of the fluorophore-binding pocket of the RNA. Further, the carbonyl oxygen of the amide group of SALAD1 is uniquely within coordination distance (3.1 Å) from the K^+^ ion of the adjacent G-quadruplex (at the precision of the current atomic coordinates; **Table S2**). Altogether, the larger interfacial area, the enhanced interaction with A22 resulting from the RNA conformational change, and the additional metal ion coordination are consistent with the improved properties of SALAD1.

### In-cell confocal imaging of Mango II RNA using the improved dye

Historically, efforts to improve fluorescent aptamers as in-cell imaging tools have focused on altering brightness, photostability, and background signal. Mango II-TO1-Biotin has been used to image RNA localization in cells via single molecule fluorescence microscopy,^13^ but only limited work has been published using confocal microscopy to image Mango systems,^25, 42^ likely due to insufficient brightness. Confocal microscopy enables the capture of high-resolution images of in-focus light, making it a powerful technique for imaging RNA fluorogenic aptamers that might otherwise display background fluorescence. HEK293T cells were transfected with the previously described^24^ mCherry-Mango II x 24 plasmid which allows both protein and RNA expression levels to be monitored in cells in the same imaging experiment. Cells were fixed and treated with 2 μM of either SALAD1 or TO1-Biotin and prepared for imaging.

**Figure 5:**
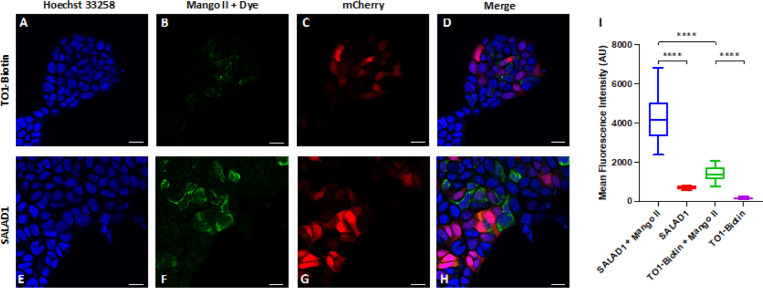
Confocal imaging of HEK293T cells transiently transfected with a plasmid expressing an mCherry-Mango II x 24 construct and treated with TO1-Biotin (A-D) or SALAD1 (E-H). Hoechst 33258 signal is blue, SALAD1 or TO1-Biotin signal is green, and mCherry signal is red. Scale bar indicates 20 micrometers. (I) Quantification of the fluorescence in B and F by mean fluorescence intensity of SALAD1 or TO1-Biotin in transfected cells (with Mango II) or non-transfected cells. Number of cells used: SALAD1 + Mango II, n=14; SALAD1, n=5; TO1-Biotin + Mango II, n=10; TO1-Biotin, n=5. ^****^ = P-value <0.0001.

In cells with stained with Hoechst dye to image nuclei (Fig. 5A and E), Mango II-containing RNA transcription (Fig. 5B and F) and mCherry expression (Fig. 5C and G) were observed via fluorescent imaging. The new SALAD1 dye visually fluoresces brighter than TO1-Biotin when bound to Mango II in cells (Figure. 5B and F). Additionally, mean fluorescence intensity is 3-fold brighter (Figure. 5I). Thus, SALAD1 as a dramatically brighter fluorophore, is suitable for confocal imaging and holds notable potential for imaging RNA in whole cells.

## CONCLUSIONS

Here, we show that the development of novel RNA-binding ligands with improved properties can be achieved through a fragment-based, structure-informed strategy. The Mango II system is a rare example of a well-characterized RNA-ligand complex and was well-suited to explore our approach. We used an SMM screening platform in concert with a curated library of fragments to discover a fragment that co-binds, non-competitively, with TO to Mango II and increases the fluorescence intensity of TO. Covalently linking the fragment to TO resulted in a dye with subnanomolar affinity for the aptamer. Our approach conceptually differs from methods that focus on chemical alteration of the chromophore, and we find that fragment linking results in unexpected and welcome improvements of diverse fluorophore properties, such as high affinity, selectivity, turn-on ratio, and fluorescence intensity.

Compared to TO and TO1-Biotin, the dye described here displays much higher fluorescence intensity in the presence of Mango II. Remarkably, SALAD1 also fluoresces brighter than the other closely related analogs lacking specific functional groups decorating the benzyl fragment, indicating that both substituents are necessary to achieve high fluorescence enhancement. The pyrazole ring in particular seems to play an important role for the turn-on and extinction coefficient values of the synthesized dyes. This finding demonstrates that our screening approach produced a fragment hit with specific properties that led to the design of the improved dye. Additionally, while all four SALAD analogs all bound tighter than TO to Mango II, crystal structures revealed that the unique binding pose of SALAD1 and associated conformational effects on the RNA may explain the unique enhanced fluorescence properties of the dye. In contrast to the other Mango-dye complex structures, SALAD1 binds to Mango II in a manner that results in a more enclosed binding site through altering the conformation of A22. Not only that, but this binding pose positions the carbonyl oxygen within bonding distance of the stabilizing potassium ion through the center of the guanine tetrad. To the best of our knowledge this is the first time a small molecule ligand has been observed to position a group within bonding distance of the potassium ion of a G4, a novel bonding interaction that could have broad implications for the design of other G4 ligands. By constraining the fluorophore in a unique pose, these new binding features contribute to both the dramatic increase in binding affinity and fluorescence intensity of SALAD1, highlighting the utility of our approach for structure-informed ligand design.

The net result of these distinctive features and the specific molecular interaction of SALAD1 is a new brighter and more practical class of RNA-activated turn-on fluorophores. Due to its bright fluorescence and low background, the “Mango-SALAD” system is well-suited for confocal imaging and is poised to create opportunities to better study RNA expression and localization. Additionally, this work stands as a powerful demonstration of the potential for structure-informed design and fragment-based discovery to develop novel ligands with improved binding affinity, selectivity, and other properties for RNA targets. Design strategies described herein have implications both for the development of improved imaging probes, including other fluorogenic RNA aptamers, as well as for medicinal chemistry efforts to develop biologically active compounds that interact with therapeutically relevant RNAs.

## Figures and Tables

**Table 1:** Photophysical properties of TO-based fluorophores

Dye	Free dye abs. max (nm)	${\text{\textbackslash{}varvec}\lambda }_{\backslash \text{varvec}e\backslash \text{varvec}x\backslash \text{varvec}c}^{\backslash \text{varvec}m\backslash \text{varvec}a\backslash \text{varvec}x}$ (nm)	${\text{\textbackslash{}varvec}\lambda }_{\backslash \text{varvec}e\backslash \text{varvec}m}^{\backslash \text{varvec}m\backslash \text{varvec}a\backslash \text{varvec}x}$ (nm)	$\begin{array}{c} \backslash \text{varvec}m\backslash \text{varvec}a\backslash \text{varvec}x\\ \backslash \text{varvec}a\backslash \text{varvec}b\backslash \text{varvec}s \end{array}$ (M^− 1^ cm^− 1^)	Turn-on (fold)	K_D_ (nM)
TO	500	510	533	53,784	643	5.9 ± 1.4
TO1-Biotin	510	510	535	77,500^a^	647	0.85 ± 0.2
SALAD1	497	511	540	45,422	514	0.69 ± 0.1
SALAD2	496	510	532	14,307	11	0.27 ± 0.03
SALAD3	497	515	541	30,235	711	0.29 ± 0.02
SALAD4	497	509	532	22,448	19	0.21 ± 0.03

a Obtained in a previous study^25^
